# Supplementary material for: Rare and highly destructive wildfires drive human migration in the U.S
Source: Nat Commun. 2024 Aug 5;15:6631. doi: 10.1038/s41467-024-50630-4 (PMC11300458; doi:10.1038/s41467-024-50630-4)
Supplement: Supplementary file 1 — Supplementary Information [file 41467_2024_50630_MOESM1_ESM.pdf]

Supplementary Information: Rare and highly destructive wildfires drive human migration in the U.S.

Contents

1 Full regression results for matched difference-in-differences models 2

2 Comparison of burn area to spatial unit area 6

3 Results of coarsened exact matching 9

1 Full regression results for matched difference-in-differences models

Table S.I.1: Wildfire Effects on Out-Migration Probability

	Full top decile			Less destructive portion			More destructive portion		
	5 Mi.	5-25 Mi.	25-50 Mi.	5 Mi.	5-25 Mi.	25-50 Mi.	5 Mi.	5-25 Mi.	25-50 Mi.
Unburned Tract	0.0440***	0.0441***	0.0438***	0.0419***	0.0468***	0.0450***	0.0451***	0.0450***	0.0438***
	(0.0008)	(0.0008)	(0.0007)	(0.0011)	(0.0011)	(0.0010)	(0.0011)	(0.0012)	(0.0009)
Burned Tract	−0.0031**	−0.0033***	−0.0025**	−0.0037*	−0.0084***	−0.0067***	−0.0027	−0.0021	−0.0011
	(0.0011)	(0.0010)	(0.0010)	(0.0017)	(0.0017)	(0.0016)	(0.0015)	(0.0015)	(0.0013)
Event Quarter	−0.0038***	−0.0017*	−0.0030***	−0.0002	0.0022	−0.0006	−0.0025	−0.0038*	−0.0044***
	(0.0010)	(0.0008)	(0.0008)	(0.0017)	(0.0015)	(0.0014)	(0.0014)	(0.0015)	(0.0012)
First Year Post-Event Quarter	−0.0013*	−0.0006	−0.0003	−0.0025*	−0.0037***	−0.0024***	0.0004	0.0026*	0.0010
	(0.0006)	(0.0006)	(0.0005)	(0.0010)	(0.0009)	(0.0007)	(0.0009)	(0.0011)	(0.0007)
Second Year Post-Event Quarter	−0.0010	−0.0016**	−0.0013**	−0.0027*	−0.0050***	−0.0021	−0.0014	0.0003	−0.0006
	(0.0010)	(0.0006)	(0.0005)	(0.0012)	(0.0009)	(0.0011)	(0.0007)	(0.0011)	(0.0006)
Burned*Event Quarter	0.0034*	0.0018	0.0034*	−0.0015	−0.0044*	−0.0019	0.0030	0.0031	0.0041*
	(0.0015)	(0.0014)	(0.0014)	(0.0022)	(0.0021)	(0.0018)	(0.0022)	(0.0020)	(0.0019)
Burned*First Year Post	0.0048***	0.0045***	0.0044***	0.0003	0.0014	0.0002	0.0047***	0.0026	0.0041***
	(0.0010)	(0.0010)	(0.0010)	(0.0013)	(0.0013)	(0.0012)	(0.0013)	(0.0014)	(0.0010)
Burned*Second Year Post	0.0004	0.0012	0.0011	0.0004	0.0022	−0.0001	0.0014	−0.0006	0.0006
	(0.0012)	(0.0009)	(0.0008)	(0.0017)	(0.0014)	(0.0016)	(0.0011)	(0.0014)	(0.0009)
Adj. R^2	0.0012	0.0007	0.0008	0.0032	0.0079	0.0025	0.0014	0.0022	0.0017
Num. obs.	86089	261240	315207	29790	111022	148761	42003	123754	163963
N Clusters	3630	8616	9749	1579	4780	5867	1873	5534	6859

$^{***}p < 0.0021$; $^{**}p < 0.01$; $^{*}p < 0.05$. *** indicates Bonferroni-adjusted p-value threshold. Models report full results from difference-in-differences models, which use weights derived from coarsened exact matching. Two-sided p-values are reported. Robust standard errors are clustered at the census tract level and shown in parentheses. Sources: Federal Reserve Bank of New York/Equifax Consumer Credit Panel and U.S. National Incident Management System/Incident Command System (1).

Table S.I.2: 2018 Camp Fire Effects on Out-Migration Probability

	Out-Migration Probability		
	5 Mi.	5-25 Mi.	25-50 Mi.
Unburned Tract	0.0339***	0.0349***	0.0355***
	(0.0033)	(0.0044)	(0.0057)
Burned Tract	0.0029	0.0000	0.0018
	(0.0053)	(0.0056)	(0.0066)
Event Quarter	0.0005	−0.0017	−0.0045
	(0.0044)	(0.0045)	(0.0061)
First Four Post-Event Quarters	0.0075***	0.0038*	0.0012
	(0.0013)	(0.0017)	(0.0017)
Second Four Post-Event Quarters	0.0006	0.0003	0.0058
	(0.0014)	(0.0060)	(0.0031)
Burned*Event Quarter	0.0535**	0.0693***	0.0694***
	(0.0188)	(0.0190)	(0.0179)
Burned*First 4 Post-Event Quarters	0.0680***	0.0833***	0.0828***
	(0.0191)	(0.0208)	(0.0182)
Burned*Second 4 Post-Event Quarters	0.0191*	0.0258*	0.0162
	(0.0088)	(0.0098)	(0.0081)
Adj. R^2	0.2891	0.2422	0.1822
Num. obs.	816	714	884
N Clusters	48	42	52

$^{***}p < 0.0021$; $^{**}p < 0.01$; $^{*}p < 0.05$. *** indicates Bonferroni-adjusted p-value threshold. Models report full results from difference-in-differences models, which use weights derived from coarsened exact matching. Two-sided p-values are reported. Robust standard errors are clustered at the census tract level and shown in parentheses. Sources: Federal Reserve Bank of New York/Equifax Consumer Credit Panel and U.S. National Incident Management System/Incident Command System (1).

Table S.I.3: Wildfire Effects on In-Migration Probability

	Full top decile			Less destructive portion			More destructive portion		
	5 Mi.	5-25 Mi.	25-50 Mi.	5 Mi.	5-25 Mi.	25-50 Mi.	5 Mi.	5-25 Mi.	25-50 Mi.
Unburned Tract	0.0500***	0.0505***	0.0502***	0.0461***	0.0526***	0.0494***	0.0508***	0.0526***	0.0498***
	(0.0012)	(0.0011)	(0.0011)	(0.0015)	(0.0016)	(0.0014)	(0.0017)	(0.0018)	(0.0016)
Burned Tract	−0.0030*	−0.0039*	−0.0027	−0.0019	−0.0077***	−0.0044	−0.0025	−0.0043	−0.0016
	(0.0015)	(0.0016)	(0.0016)	(0.0023)	(0.0025)	(0.0023)	(0.0022)	(0.0022)	(0.0020)
Event Quarter	−0.0061***	−0.0055***	−0.0072***	−0.0024	−0.0013	−0.0045***	−0.0065***	−0.0096***	−0.0069***
	(0.0013)	(0.0010)	(0.0010)	(0.0016)	(0.0015)	(0.0011)	(0.0021)	(0.0015)	(0.0014)
First Year Post-Event Quarter	−0.0038**	−0.0034***	−0.0034***	−0.0037*	−0.0065***	−0.0038***	−0.0021	0.0005	0.0000
	(0.0013)	(0.0009)	(0.0010)	(0.0014)	(0.0014)	(0.0009)	(0.0016)	(0.0012)	(0.0010)
Second Year Post-Event Quarter	−0.0055***	−0.0049***	−0.0050***	−0.0027	−0.0051*	−0.0043***	−0.0051***	−0.0057***	−0.0035***
	(0.0010)	(0.0010)	(0.0010)	(0.0021)	(0.0022)	(0.0012)	(0.0015)	(0.0013)	(0.0008)
Burned*Event Quarter	−0.0004	−0.0006	−0.0001	−0.0013	−0.0024	−0.0004	−0.0010	0.0016	−0.0011
	(0.0017)	(0.0016)	(0.0016)	(0.0022)	(0.0023)	(0.0020)	(0.0026)	(0.0021)	(0.0020)
Burned*First Year Post	0.0023	0.0023	0.0020	−0.0006	0.0013	−0.0010	0.0024	−0.0004	0.0002
	(0.0016)	(0.0013)	(0.0014)	(0.0021)	(0.0022)	(0.0020)	(0.0021)	(0.0015)	(0.0015)
Burned*Second Year Post	0.0009	0.0010	0.0010	−0.0013	−0.0000	−0.0001	0.0016	0.0016	−0.0002
	(0.0013)	(0.0014)	(0.0014)	(0.0029)	(0.0030)	(0.0025)	(0.0018)	(0.0016)	(0.0012)
Adj. R^2	0.0033	0.0027	0.0031	0.0021	0.0060	0.0039	0.0028	0.0051	0.0026
Num. obs.	86126	261273	315255	29820	111045	148778	42012	123762	163995
N Clusters	3630	8616	9752	1579	4780	5867	1873	5534	6859

$^{***}p < 0.0021$; $^{**}p < 0.01$; $^{*}p < 0.05$. *** indicates Bonferroni-adjusted p-value threshold. Models report full results from difference-in-differences models, which use weights derived from coarsened exact matching. Two-sided p-values are reported. Robust standard errors are clustered at the census tract level and shown in parentheses. Sources: Federal Reserve Bank of New York/Equifax Consumer Credit Panel and U.S. National Incident Management System/Incident Command System (1).

Table S.I.4: 2018 Camp Fire: Wildfire Effects on In-Migration Probability

	In-Migration Probability		
	5 Mi.	5-25 Mi.	25-50 Mi.
Unburned Tract	0.0380***	0.0406***	0.0364***
	(0.0024)	(0.0034)	(0.0037)
Burned Tract	0.0029	−0.0004	0.0047
	(0.0039)	(0.0045)	(0.0046)
Event Quarter	0.0074	0.0089	−0.0162*
	(0.0055)	(0.0045)	(0.0079)
First Four Post-Event Quarters	0.0067	−0.0001	0.0029
	(0.0052)	(0.0057)	(0.0029)
Second Four Post-Event Quarters	−0.0030	−0.0039	0.0015
	(0.0021)	(0.0020)	(0.0035)
Burned*Event Quarter	0.0060	0.0060	0.0295**
	(0.0089)	(0.0079)	(0.0097)
Burned*First 4 Post-Event Quarters	0.0131	0.0169*	0.0179**
	(0.0074)	(0.0082)	(0.0065)
Burned*Second 4 Post-Event Quarters	0.0103*	0.0119*	0.0060
	(0.0049)	(0.0049)	(0.0052)
Adj. R^2	0.0577	0.0299	0.0660
Num. obs.	816	714	884
N Clusters	48	42	52

$^{***}p < 0.0021$; $^{**}p < 0.01$; $^{*}p < 0.05$. *** indicates Bonferroni-adjusted p-value threshold. Models report full results from difference-in-differences models, which use weights derived from coarsened exact matching. Two-sided p-values are reported. Robust standard errors are clustered at the census tract level and shown in parentheses. Sources: Federal Reserve Bank of New York/Equifax Consumer Credit Panel and U.S. National Incident Management System/Incident Command System (1).

2 Comparison of burn area to spatial unit area

The population of wildfires which we examine ranges considerably in the amount of land burned per incident (Figure A1). The smallest in this population burned less than one acre, while the largest, the 2020 August Complex Fire, burned over one million acres. This range of burn area sizes raises the question of which spatial unit is best suited for analyzing the impacts of destructive wildfires, and indicates that no one unit will perfectly align with the spatial scale of all incidents. For this study, we utilize census tracts, which we selected because this unit offers the closest approximation to neighborhoods (2) and because it more closely matches the spatial scale of wildfires compared to counties, the unit used in comparable existing research. In cases in which a fire burned an area larger than the tract in which it ignited, our spatial allocation procedure combines all affected tracts into one spatial cluster for analysis.

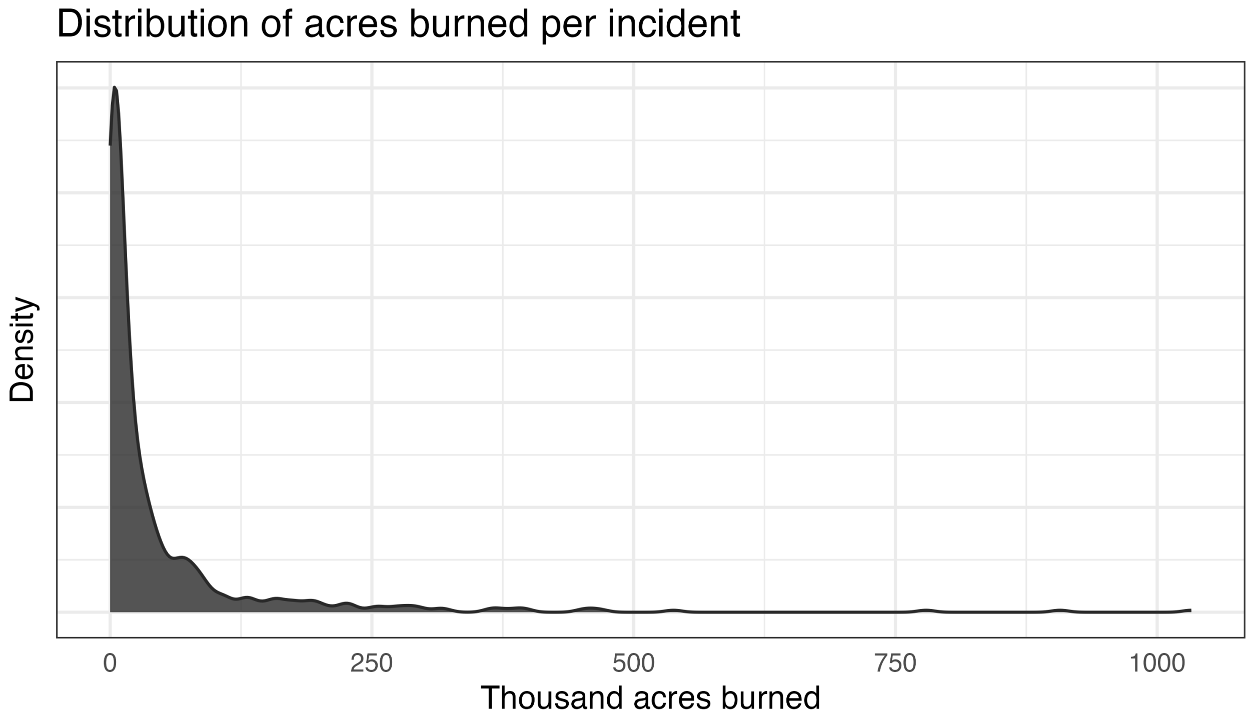

Figure S.I.1. Distribution of acres burned per incident. Distribution of acres burned per incident among the 529 most destructive wildfire incidents in the contiguous U.S. examined in the main analysis. Source: U.S. National Incident Management System/Incident Command System (1).

Previous quantitative research on wildfires and population change has used the county scale (3; 4; 5), which is in keeping with research designs used to study hurricane impacts (6; 7; 8). However, at first glance, the spatial scale of wildfire footprints is generally much smaller than that of hurricanes, suggesting that counties may be substantially larger than the spatial regions that wildfires affect within them. To assess whether this is the case, we performed a supplementary analysis of the spatial scale of the population of destructive wildfires included in the manuscript's primary analysis and which include point of origin details in the ICS data (N=525). We calculate the ratio of final acres burned per incident (reported in the ICS data) to the total acres of the spatial unit in which that incident ignited (we term "burn area to county ratio" and "burn area to tract ratio"). This ratio gives a rough approximation of the proportion of a spatial unit burned.

Results suggest that highly destructive wildfire burn footprints are generally smaller in area than both the county and the tract in which they ignited (Figure A2, Tables A5 and A6); this difference in size is especially pronounced between wildfires and counties. Among our population of highly destructive wildfires in the contiguous U.S., 91.3% of incidents burned less than 10% of the overall land area of the county in which they ignited. This estimate is 60.3% for census tracts. These trends indicate that sub-county scale units are preferable for research designs focused on direct wildfire burn impacts.

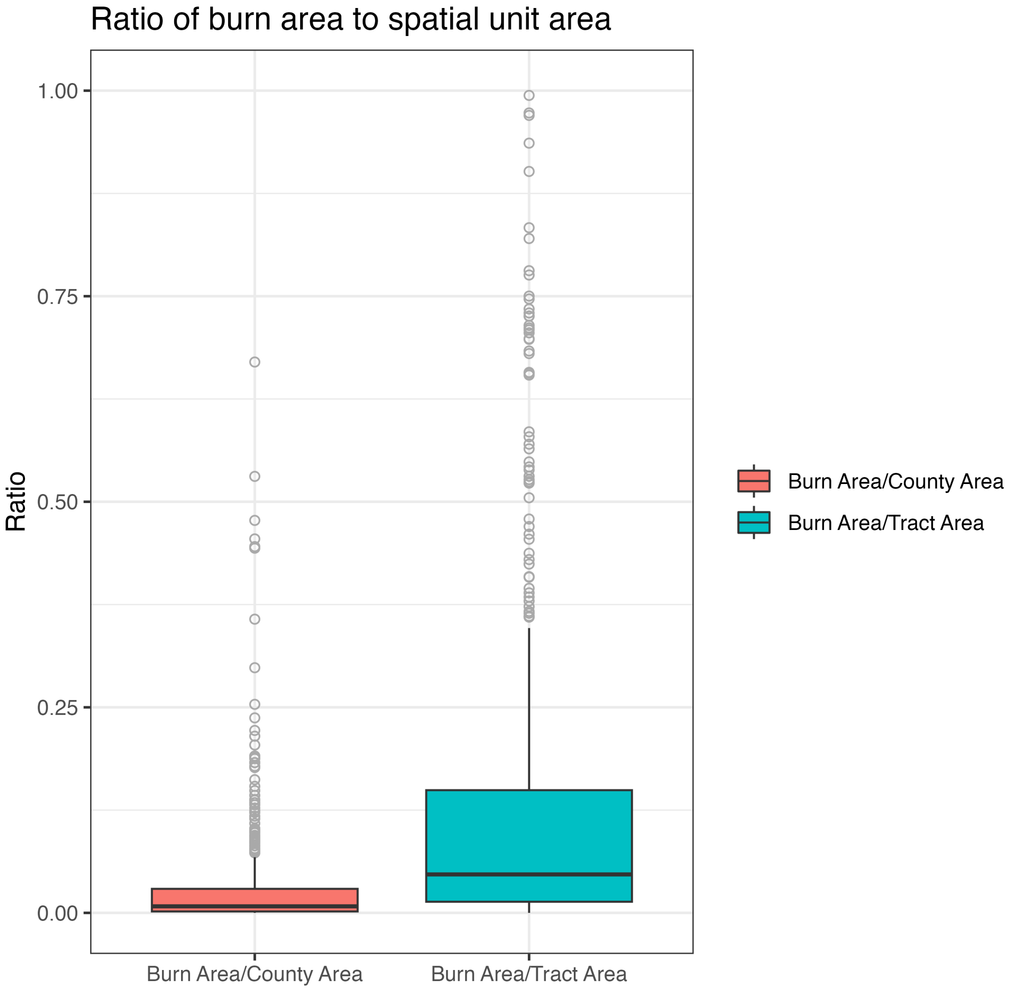

Figure S.I.2. Distribution of burn area to county and burn area to tract ratios. For display purposes, the figure removes incidents with ratios greater than one. Of the incidents examined, two had burn area to county ratios greater than one and 48 had burn area to tract ratios greater than one. Source: U.S. National Incident Management System/Incident Command System (1).

Table S.I.5

Burned area to county area ratio	Incidents	Percent of incidents
(0.0 - 0.25]	514	97.9%
(0.25 - 0.5]	7	1.3%
(0.5 - 0.75]	2	0.4%
(0.75 - 1.0]	0	0.0%
>1.0	2	0.4%

Note: Comparison of destructive wildfire burn area relative to county of origin. N = 525, which includes the population incidents analyzed in main manuscript, with four incidents removed due to missing point of origin. Source: U.S. National Incident Management System/Incident Command System (1).

Table S.I.6

Burned area to tract area ratio	Incidents	Percent of incidents
(0.0 - 0.25]	393	74.9%
(0.25 - 0.5]	46	8.8%
(0.5 - 0.75]	28	5.3%
(0.75 - 1.0]	10	1.9%
>1.0	48	9.1%

Note: Comparison of destructive wildfire burn area relative to tract of origin. N = 525, which includes the population incidents analyzed in main manuscript, with four incidents removed due to missing point of origin. Source: U.S. National Incident Management System/Incident Command System (1).

3 Results of coarsened exact matching

Plotted points show absolute standardized differences in means across relevant covariates (9; 10; 11) between treated (burned) and control (unburned neighboring) among the top 10% of all destructive wildfires between 1999 and 2020. Red points show differences in means where control tracts are selected only based on distance criteria and without matching. Blue points show differences in means across relevant covariates between treated and control tracts after CEM matching. The vertical dashed line indicates where absolute standardized mean difference = 0.1, a conservative threshold for evaluating covariate balance. Prior to matching, most covariates were unbalanced between treatment and control tracts, whereas after matching, nearly all covariates are well-balanced.

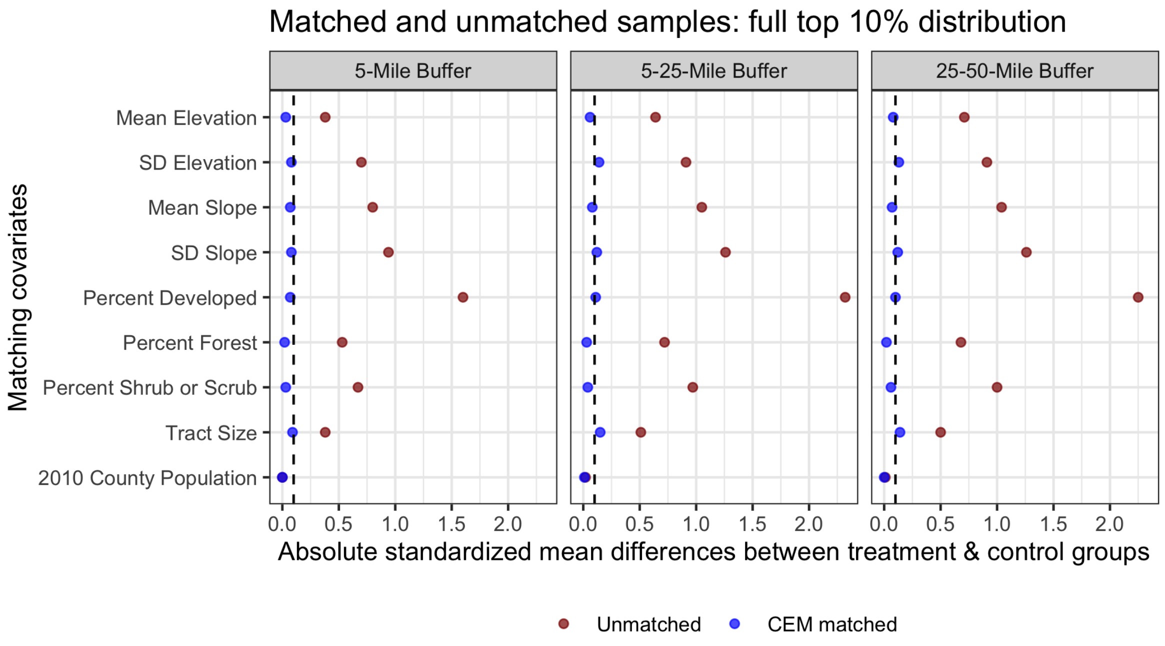

Figure S.I.3. Absolute standardized mean differences between treated and untreated tracts for the full decile of most destructive wildfires. Red dots show unmatched mean differences and blue dots show matched mean differences. Sources: U.S. National Incident Management System/Incident Command System (1), 90m SRTM digital elevation map (9), 2019 National Land Cover Database (10), and 2010 county-level population estimates (11).

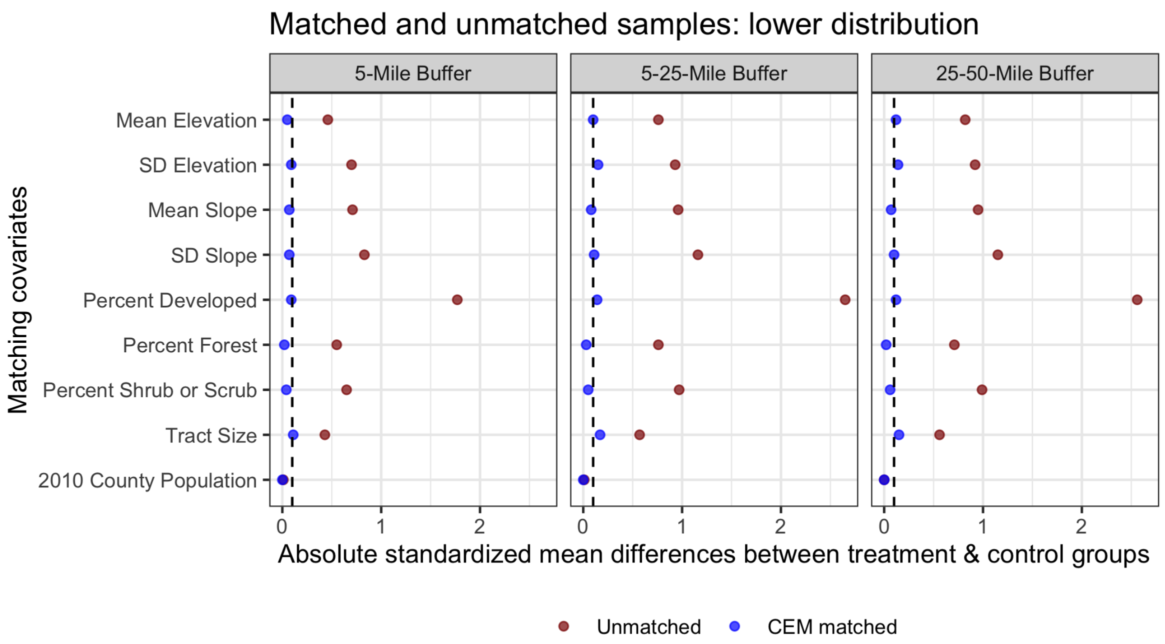

Figure S.I.4. Absolute standardized mean differences between treated and untreated tracts for the less destructive portion of the wildfire distribution. Red dots show unmatched mean differences and blue dots show matched mean differences. Sources: U.S. National Incident Management System/Incident Command System (1), 90m SRTM digital elevation map (9), 2019 National Land Cover Database (10), and 2010 county-level population estimates (11).

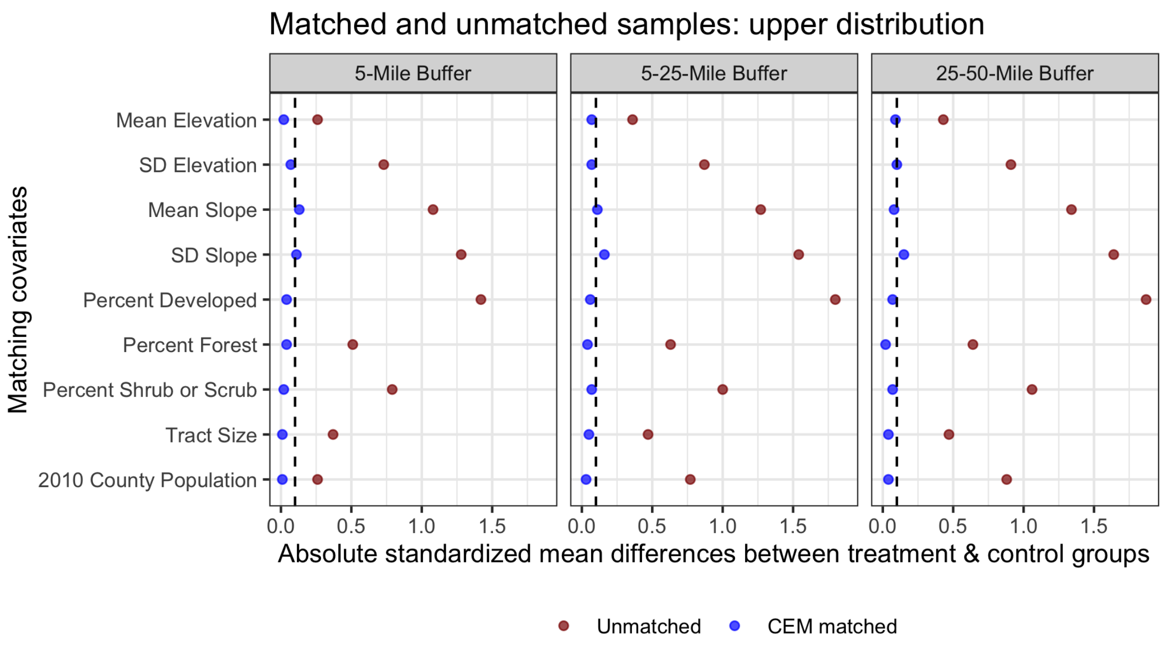

Figure S.I.5. Absolute standardized mean differences between treated and untreated tracts for the more destructive portion of the wildfire distribution. Red dots show unmatched mean differences and blue dots show matched mean differences. Sources: U.S. National Incident Management System/Incident Command System (1), 90m SRTM digital elevation map (9), 2019 National Land Cover Database (10), and 2010 county-level population estimates (11).

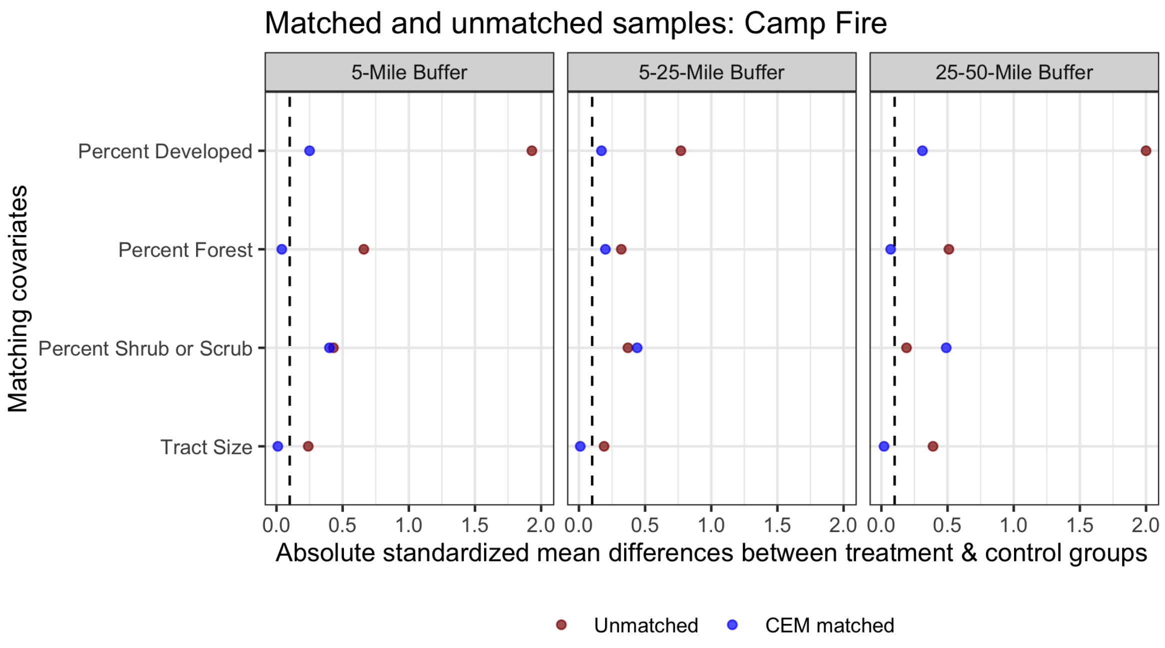

Figure S.I.6. Absolute standardized mean differences between treated and untreated tracts for the Camp Fire. Red dots show unmatched mean differences and blue dots show matched mean differences. Sources: U.S. National Incident Management System/Incident Command System (1), 90m SRTM digital elevation map (9), 2019 National Land Cover Database (10), and 2010 county-level population estimates (11).

References

[1] St. Denis, L. A. *et al.* All-hazards dataset mined from the US National Incident Management System 1999–2020. *Scientific Data* **10**, 112 (2023). URL https://www.nature.com/articles/s41597-023-01955-0. Number: 1 Publisher: Nature Publishing Group.

[2] Bureau, U. C. Glossary (2022). URL https://www.census.gov/programs-surveys/geography/about/glossary.html. Section: Government.

[3] Winkler, R. L. & Rouleau, M. D. Amenities or disamenities? Estimating the impacts of extreme heat and wildfire on domestic US migration. *Population and Environment* (2020). URL https://doi.org/10.1007/s11111-020-00364-4.

[4] DeWaard, J. *et al.* Migration as a Vector of Economic Losses From Disaster-Affected Areas in the United States. *Demography* **60**, 173–199 (2023).

[5] Clark, M. B., Nkonya, E. & Galford, G. L. Flocking to fire: How climate and natural hazards shape human migration across the United States. *Frontiers in Human Dynamics* **4** (2022). URL https://www.frontiersin.org/articles/10.3389/fhumd.2022.886545.

[6] Curtis, K. J., Fussell, E. & DeWaard, J. Recovery Migration After Hurricanes Katrina and Rita: Spatial Concentration and Intensification in the Migration System. *Demography* **52**, 1269–1293 (2015). URL https://doi.org/10.1007/s13524-015-0400-7.

[7] Fussell, E., Curtis, K. J. & DeWaard, J. Recovery migration to the City of New Orleans after Hurricane Katrina: a migration systems approach. *Population and Environment* **35**, 305–322 (2014). URL https://doi.org/10.1007/s11111-014-0204-5.

[8] Fussell, E., DeWaard, J. & Curtis, K. J. Environmental migration as short- or long-term differences from a trend: A case study of Hurricanes Katrina and Rita effects on out-migration in the Gulf of Mexico. *International Migration* (2022). URL https://onlinelibrary.wiley.com/doi/10.1111/imig.13101.

[9] Jarvis, A., Reuter, H. I., Nelson, A. & Guevara, E. Hole-filled SRTM for the globe Version 4. *available from the CGIAR-CSI SRTM 90m Database (http://srtm. csi. cgiar. org)* **15**, 5 (2008).

[10] Dewitz, J. & Survey, U. G. National Land Cover Database (NLCD) 2019 Products (ver. 2.0, June 2021) (2021).

[11] of Agriculture Economic Research Service, U. S. D. Rural-Urban Commuting Area Codes (2020). URL https://www.ers.usda.gov/data-products/rural-urban-commuting-area-codes.aspx.
