# Supplementary material for: Rare and highly destructive wildfires drive human migration in the U.S
Source: Nat Commun. 2024 Aug 5;15:6631. doi: 10.1038/s41467-024-50630-4 (PMC11300458; doi:10.1038/s41467-024-50630-4)
Supplement: Supplementary file 3 — Reporting Summary [file 41467_2024_50630_MOESM3_ESM.pdf]

nature portfolio

Corresponding author(s): Kathryn McConnell

Last updated by author(s): June 13, 2024

Reporting Summary

Nature Portfolio wishes to improve the reproducibility of the work that we publish. This form provides structure for consistency and transparency in reporting. For further information on Nature Portfolio policies, see our Editorial Policies and the Editorial Policy Checklist.

Statistics

For all statistical analyses, confirm that the following items are present in the figure legend, table legend, main text, or Methods section.

n/a	Confirmed	
☐	☒	The exact sample size (*n*) for each experimental group/condition, given as a discrete number and unit of measurement
☐	☒	A statement on whether measurements were taken from distinct samples or whether the same sample was measured repeatedly
☐	☒	The statistical test(s) used AND whether they are one- or two-sided<br>*Only common tests should be described solely by name; describe more complex techniques in the Methods section.*
☐	☒	A description of all covariates tested
☐	☒	A description of any assumptions or corrections, such as tests of normality and adjustment for multiple comparisons
☐	☒	A full description of the statistical parameters including central tendency (e.g. means) or other basic estimates (e.g. regression coefficient) AND variation (e.g. standard deviation) or associated estimates of uncertainty (e.g. confidence intervals)
☐	☒	For null hypothesis testing, the test statistic (e.g. *F*, *t*, *r*) with confidence intervals, effect sizes, degrees of freedom and *P* value noted<br>*Give P values as exact values whenever suitable.*
☒	☐	For Bayesian analysis, information on the choice of priors and Markov chain Monte Carlo settings
☒	☐	For hierarchical and complex designs, identification of the appropriate level for tests and full reporting of outcomes
☒	☐	Estimates of effect sizes (e.g. Cohen's *d*, Pearson's *r*), indicating how they were calculated

Our web collection on statistics for biologists contains articles on many of the points above.

Software and code

Policy information about availability of computer code

Data collection	Wildfire data and covariates used for coarsened exact matching are publicly available and documented in full in references 33, 54, 55, and 57. Migration measures were obtained from the Federal Reserve Bank of New York/Equifax Consumer Credit Panel (CCP).
Data analysis	Primary analyses were conducted using R statistical software versions 4.3.3 and 4.4.0. Land cover, elevation, and slope statistics used for coarsened exact matching were processed in Google Earth Engine. Codes used to process public data are available at https://osf.io/xa39e/. The following R packages were used for the manuscript's analysis: classInt (0.4.10), cowplot (1.1.3), dint (2.1.4), dplyr (1.1.4), estimatr (1.0.4), ggthemes (5.1.0), lubridate (1.9.3), MatchIt (4.5.5), purrr (1.0.2), sf (1.0.16), stringr (1.5.1), texreg (1.39.3), tigris (2.1), and tidyverse (2.0.0).

For manuscripts utilizing custom algorithms or software that are central to the research but not yet described in published literature, software must be made available to editors and reviewers. We strongly encourage code deposition in a community repository (e.g. GitHub). See the Nature Portfolio guidelines for submitting code & software for further information.

Data

Policy information about availability of data

All manuscripts must include a data availability statement. This statement should provide the following information, where applicable:

- Accession codes, unique identifiers, or web links for publicly available datasets
- A description of any restrictions on data availability
- For clinical datasets or third party data, please ensure that the statement adheres to our policy

Wildfire administrative data from the U.S. National Incident Management System/Incident Command System were published by St. Denis et al. 2023 (https://www.nature.com/articles/s41597-023-01955-0). Wildfire footprints were accessed through the Monitoring Trends in Burn Severity database (https://www.mtbs.gov/) and the Fire Events Delineation database, published by Balch et al. 2020 (https://www.mdpi.com/2072-4292/12/21/3498). Covariate data used for coarsened exact matching were accessed through the CGIAR-CSI SRTM 90m Database (https://developers.google.com/earth-engine/datasets/catalog/CGIAR_SRTM90_V4), the National Land Cover Database (https://developers.google.com/earth-engine/datasets/catalog/USGS_NLCD_RELEASES_2019_REL_NLCD), and the United States Department of Agriculture Economic Research Service (https://www.ers.usda.gov/data-products/rural-urban-continuum-codes.aspx). Census tract boundaries were obtained from NHGIS (https://data2.nhgis.org/main). Migration measures were obtained from the Federal Reserve Bank of New York/Equifax Consumer Credit Panel. These are proprietary data that our Federal Reserve affiliated co-author accessed through an institutional Data Use Agreement. They are not publicly available and we are not permitted to share them. Source data for figures are provided with this paper. Codes developed to process the publicly available data listed above are available through OSF at https://osf.io/xa39e/.

Research involving human participants, their data, or biological material

Policy information about studies with human participants or human data. See also policy information about sex, gender (identity/presentation), and sexual orientation and race, ethnicity and racism.

Reporting on sex and gender	This study does not constitute human subjects research, as it is based on secondary data with no personally identifying information.
Reporting on race, ethnicity, or other socially relevant groupings	Population characteristics such as race and ethnicity are not available.
Population characteristics	See above.
Recruitment	No participants were recruited for this study.
Ethics oversight	N/A

Note that full information on the approval of the study protocol must also be provided in the manuscript.

Field-specific reporting

Please select the one below that is the best fit for your research. If you are not sure, read the appropriate sections before making your selection.

☐ Life sciences ☒ Behavioural & social sciences ☐ Ecological, evolutionary & environmental sciences

For a reference copy of the document with all sections, see nature.com/documents/nr-reporting-summary-flat.pdf

Behavioural & social sciences study design

All studies must disclose on these points even when the disclosure is negative.

Study description	This quantitative study conducts a difference-in-differences regression analysis to investigate the effects of wildfire building destruction on in-migration and out-migration probabilities at the census tract scale.
Research sample	Our research sample includes all census tracts that were burned by the top 10% most destructive wildfires in the contiguous United States between 1999 and 2020. These burned tracts are compared to neighboring, unburned tracts within 0-5, 5-25, and 25-50 mile buffers from the burned tracts. For these tracts, we aggregate migration measures from the Federal Reserve Bank of New York/Equifax Consumer Credit Panel (CCP), which is a five percent random sample drawn from credit histories maintained by Equifax. The CCP does not include demographic information on this sample, such as sex, race, ethnicity, or nativity. The CCP migration data only include residents with a Social Security Number (SSN) and a credit history, and therefore are not necessarily representative of the full U.S. population in all places.
Sampling strategy	No recruitment was used for this study. For migration measures, we utilized the Federal Reserve Bank of New York/Equifax Consumer Credit Panel (CCP), which is a five percent random sample drawn from the credit histories maintained by Equifax.
Data collection	No data were collected for this study. We utilized publicly available environmental data and migration measures from the Federal Reserve Bank of New York/Equifax Consumer Credit Panel (CCP).

Timing	Data were used from the years 1999 through 2022.
Data exclusions	In- and out-migration probabilities varied more widely in tracts with small populations, which is in part due to the data's small sample size within these tracts. To minimize the influence of these outliers, observations with an in-migration probability greater than two standard deviations above the full dataset's mean in-migration were removed and observations with an out-migration probability greater than the maximum quarterly out-migration observed following the 2018 Camp Fire were removed.
Non-participation	No primary data were collected for this study.
Randomization	The observations in the Federal Reserve Bank of New York/Equifax Consumer Credit Panel (CCP) are a five percent random sample drawn from the credit histories maintained by Equifax.

Reporting for specific materials, systems and methods

We require information from authors about some types of materials, experimental systems and methods used in many studies. Here, indicate whether each material, system or method listed is relevant to your study. If you are not sure if a list item applies to your research, read the appropriate section before selecting a response.

Materials & experimental systems

n/a	Involved in the study
☒	☐ Antibodies
☒	☐ Eukaryotic cell lines
☒	☐ Palaeontology and archaeology
☒	☐ Animals and other organisms
☒	☐ Clinical data
☒	☐ Dual use research of concern
☒	☐ Plants

Methods

n/a	Involved in the study
☒	☐ ChIP-seq
☒	☐ Flow cytometry
☒	☐ MRI-based neuroimaging

Plants

Seed stocks	*Report on the source of all seed stocks or other plant material used. If applicable, state the seed stock centre and catalogue number. If plant specimens were collected from the field, describe the collection location, date and sampling procedures.*
Novel plant genotypes	*Describe the methods by which all novel plant genotypes were produced. This includes those generated by transgenic approaches, gene editing, chemical/radiation-based mutagenesis and hybridization. For transgenic lines, describe the transformation method, the number of independent lines analyzed and the generation upon which experiments were performed. For gene-edited lines, describe the editor used, the endogenous sequence targeted for editing, the targeting guide RNA sequence (if applicable) and how the editor was applied.*
Authentication	*Describe any authentication procedures for each seed stock used or novel genotype generated. Describe any experiments used to assess the effect of a mutation and, where applicable, how potential secondary effects (e.g. second site T-DNA insertions, mosiacism, off-target gene editing) were examined.*
